# Evidence of disruption of Si-rich microstructure in engineering-lightweight Al–12.2at.%Si alloy melt above liquidus temperature

**DOI:** 10.1038/s41598-020-69972-2

**Published:** 2020-07-31

**Authors:** Xixi Dong, Peijie Li, Sajjad Amirkhanlou, Shouxun Ji, Pjotr S. Popel, Ulf Dahlborg, Monique Calvo-Dahlborg

**Affiliations:** 1 Brunel Centre for Advanced Solidification Technology (BCAST), Institute of Materials and Manufacturing, Brunel University London, Uxbridge, UB8 3PH UK; 2 Department of Mechanical Engineering, Tsinghua University, Beijing, 100084 China; 3 Department of Materials, University of Oxford, Oxford, OX1 3PH UK; 4 Ural State Pedagogical University, Ekaterinburg, Russia 620151; 5 GPM, CNRS-UMR6634, University of Rouen Normandie, Campus Madrillet, BP12, 76801 Saint-Etienne du Rouvray, France

**Keywords:** Engineering, Materials science

## Abstract

The exploration of microstructures in high temperature alloy melts is important for manufacturing of metallic components but extremely challenging. Here, we report experimental evidence of the disruption of Si-rich microstructure in engineering-lightweight Al–12.2at.%Si alloy melt at 1100 °C, via melt-spinning (MS) of Al_1−x_Si_x_ (x = 0.03,0.07,0.122,0.2) alloy melts from different initial melt temperatures, 800 °C and 1100 °C, under the super-high cooling rate of ~ 10^6^ °C/s, in cooperation with the small angle neutron scattering (SANS) measurement. Si particles in 1100 °C MS alloys are abnormally smaller and increased in number at Al–12.2at.%Si, compared with 800 °C MS alloys, which demonstrates the disruption of Si-rich microstructure in Al–12.2at.%Si alloy melt at 1100 °C. SANS experiment verifies that large quantities of small (0–10 nm) Si-rich microstructures and small quantities of large (10–240 nm) Si-rich microstructures exist in Al–12.2at.%Si alloy melt, and the large Si-rich microstructures disrupt into small Si-rich microstructures with increasing of melt temperature from 800 to 1100 °C. Microstructure analysis of the MS alloys indicates that the large Si-rich microstructures in Al–12.2at.%Si alloy melt are probably aggregates comprising multiple small Si-rich microstructures. This work also provides a pathway for the exploration of microstructures in other high temperature alloy melts.

## Introduction

The structural materials especially metallic alloys are basic support of modern society^1,2^. Most of the metallic alloy components are manufactured from the initial melt state via the metallurgy and casting route, and the microstructure in alloy melts will affect the subsequent solidification and mechanical properties of metallic components^3–7^. However, the exploration of microstructure in alloy melts is always extraordinary challenging, due to the high temperature of alloy melts that usually ranges from several hundreds to ~ 3500 °C.

The Al–Si based alloys are important lightweight engineering alloys in automotive, aerospace and other industries, which constitute ~ 90 % of all aluminium shape castings^8–14^. As a special system, Al–Si alloy comprises two elements with a large melting point discrepancy at 754 °C (Al: 660 °C, Si: 1414 °C), which provides theoretical possibility for the existence of Si-rich microstructure in the molten state^15^. In addition, the measurement of irreversible changes in physical properties such as density during heating and cooling cycles supported the microstructure evolution in Al–Si alloy melts with changing of melt temperature^16^, however, we can hardly get information of the detail microstructure evolution in the Al–Si alloy melts from the measurement of physical properties.

X-ray diffraction (XRD) was applied to study the microstructure in Al–Si alloy melts^17–19^, and diffraction can reflect the atomic scale local short-range order structure information in Al–Si alloy melts such as the statistical mean first or second nearest neighbor atomic distance, as the measured intensity of diffraction is proportional to the scattering length of individual atomic nuclei. Unfortunately, it is hard to reveal the microstructure that is larger than the atomic scale in Al–Si alloy melts under diffraction. Molecular dynamics (MD) simulations were also performed to study the microstructure in Al–Si alloy melts^20–22^, and scarce information on the nanoscale microstructure was obtained by MD simulations, due to the limited number of atoms available to be calculated. In recent years, the technical advances in X-ray synchrotron radiation and imaging^23–26^ have made the direct imaging of micron-scale structure such as the growing dendrite in alloy melts available during the solidification process, but the direct imaging of nanoscale microstructure in alloy melts is still fiction technically. However, this does not mean that no way for the unveiling of the mystery of the detail microstructure evolution in Al–Si alloy melts that has been long-standing blind spot of science.

In the present work, we report experimental evidence of the disruption of Si-rich microstructure in the Al–12.2at.%Si alloy melt above liquidus temperature, via the melt-spinning of a serials of Al_1−x_Si_x_ (x = 0.03,0.07,0.122,0.2) alloy melts from two different initial melt temperatures, 800 °C and 1100 °C, under the super-high cooling rate of ~ 10^6^ °C/s, in cooperation with the small angle neutron scattering measurement of the Al–12.2at.%Si alloy melt.

## Results

### Abnormal decrease of Si particle size in 1100 °C MS Al–12.2at.%Si alloy

#### XRD analysis of MS Al–Si alloys

**Figure 1:**
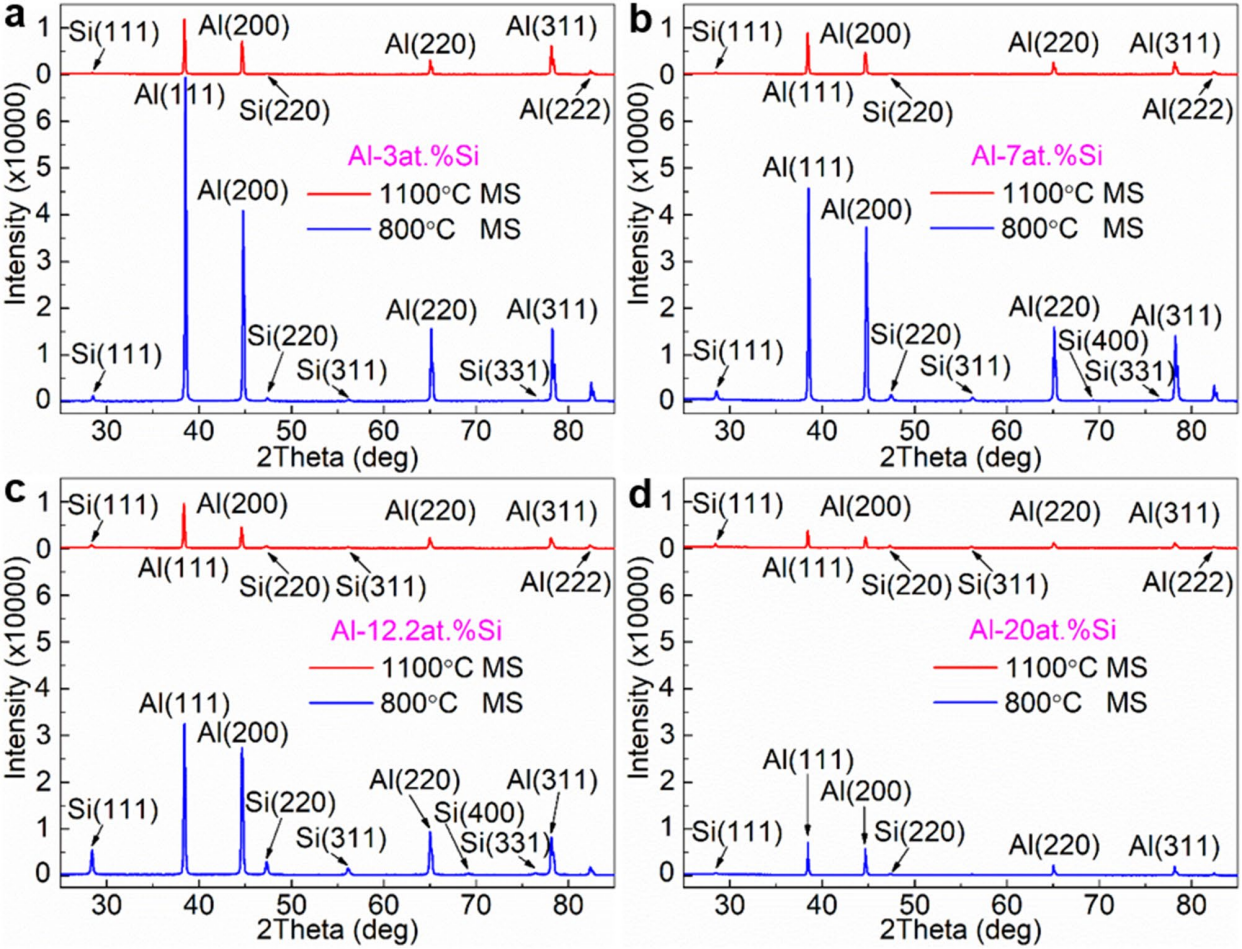
X-ray diffraction patterns of the Al–Si melt-spinning alloys rapid solidified from melts at 800 °C and 1100 °C. (**a**) Al–3at.%Si melt-spinning alloy; (**b**) Al–7at.%Si melt-spinning alloy; (**c**) Al–12.2at.%Si melt-spinning alloy; (**d**) Al–20at.%Si melt-spinning alloy.

Figure 1a–d show the XRD patterns of the 800 °C and 1100 °C MS Al–3at.%Si, Al–7at.%Si, Al–12.2at.%Si and Al–20at.%Si alloys, respectively. The XRD patterns of all the investigated MS Al–Si alloys are present in the peaks of Al phase and Si phase, indicating that the microstructure of the MS Al–Si alloys is composed of the Al matrix phase and Si phase. The XRD intensity of the Al(111) main peak of both 800 °C and 1100 °C MS Al–3at.%Si, Al–7at.%Si, Al–12.2at.%Si and Al–20at.%Si alloys decreases monotonically with the increase of Si content, except the 1100 °C MS Al–12.2at.%Si alloy whose Al(111) peak intensity shows an abnormal backing increase, when compared with the Al(111) peak intensity of the 1100 °C MS Al–7at.%Si alloy. The volume fraction of Al matrix phase that takes the majority in the investigated MS Al–Si alloys decreases with increasing Si content, and there is a big decrease in the volume fraction of the Al matrix phase from 12.2at.%Si to 20at.%Si, which might lead to the general decrease of the XRD intensity of Al with increasing Si content and the small XRD intensity of Al at the MS Al–20at.%Si alloys.

#### SEM analysis of MS Al–Si alloys

**Figure 2:**
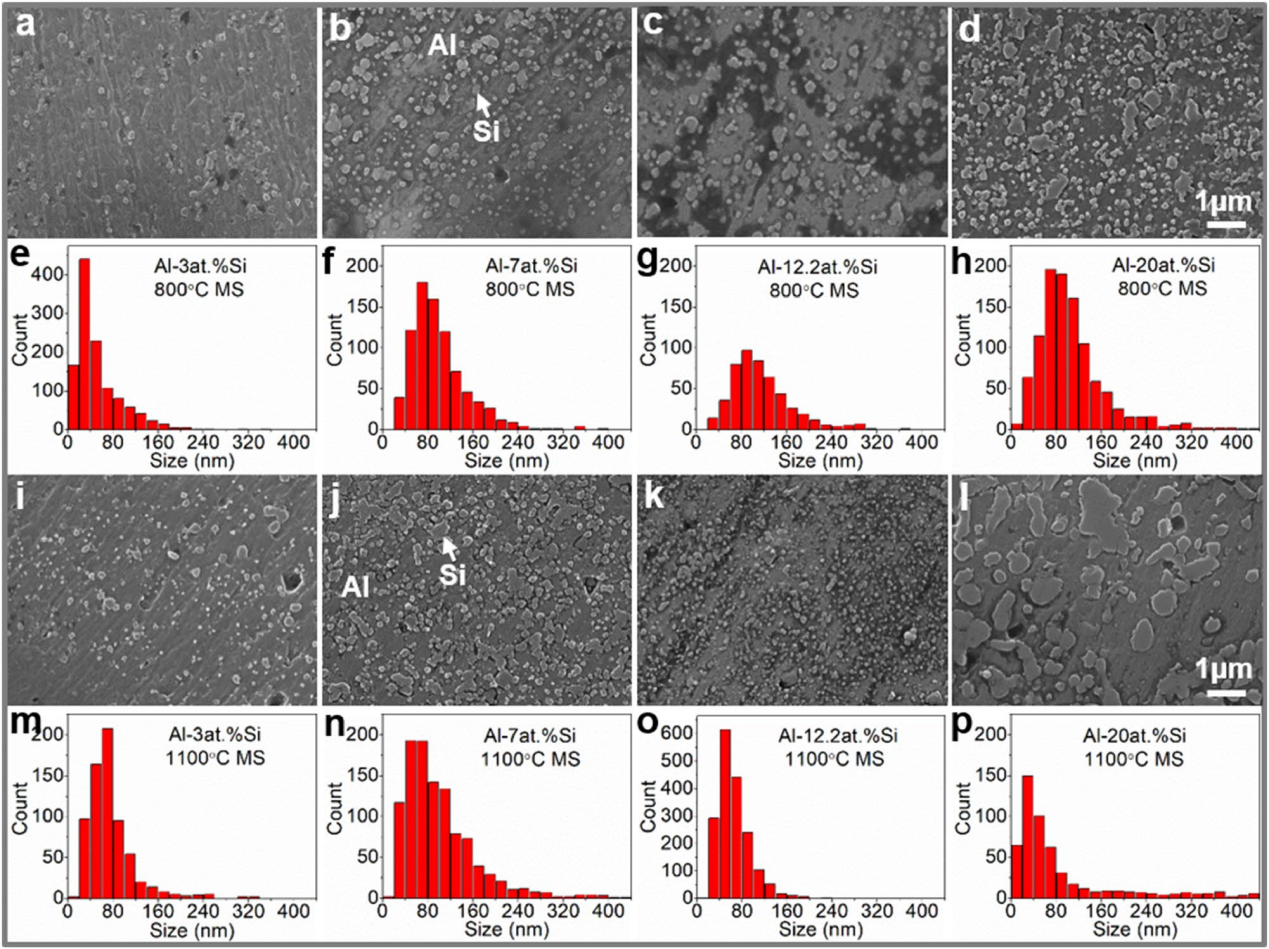
Medium magnification (×15 k) SEM micrographs taken from the cross-section center of the Al–Si melt-spinning alloy ribbons and corresponding statistical size distribution of Si particles in the Al–Si melt-spinning alloy ribbons rapid solidified from melts at (**a**–**h**) 800 °C and (**i**–**p**) 1100 °C: (**a**, **e**, **i**, **m**) Al–3at.%Si; (**b**, **f**, **j**, **n**) Al–7at.%Si; (**c**, **g**, **k**, **o**) Al–12.2at.%Si; (**d**, **h**, **l**, **p**) Al–20at.%Si.

Figure 2a–d present the medium magnification (×15 k) SEM micrographs taken from the center of the cross-section of the 800 °C MS Al–3at.%Si, Al–7at.%Si, Al–12.2at.%Si and Al–20at.%Si alloy ribbons, sequentially, and Fig. 2e–h show the corresponding statistical size distribution of Si particles in Fig. 2a–d. Si particles in the 800 °C MS Al–Si alloys show a near lognormal size distribution, with particle size ranges of (0, 220 nm), (0, 260 nm), (0, 320 nm) and (0, 440 nm) for the Al–3at.%Si, Al–7at.%Si, Al–12.2at.%Si and Al–20at.%Si MS alloys, respectively. Figure 2i–l present the medium magnification (×15 k) SEM micrographs taken from the center of the cross-section of the 1100 °C MS Al–3at.%Si, Al–7at.%Si, Al–12.2at.%Si and Al–20at.%Si alloy ribbons, sequentially, and Fig. 2m–p show the corresponding statistical size distribution of Si particles in Fig. 2i–l. Si particles in 1100 °C MS Al–Si alloys also show a near lognormal size distribution, with particle size ranges of (0, 340 nm), (0, 400 nm), (0, 200 nm) and (0, 440 nm) for the Al–3at.%Si, Al–7at.%Si, Al–12.2at.%Si and Al–20at.%Si MS alloys, separately. The size of Si particles in the 800 °C MS Al–Si alloys increases monotonically with the increase of Si content. Different from the 800 °C MS condition, with increasing Si content, the size of Si particles in the 1100 °C MS Al–Si alloys increases in a non-monotonic way with an abnormal decrease occurs at Al–12.2at.%Si. Simultaneously, the abnormal increase of the number of Si particles was observed at Al–12.2at.%Si for the 1100 °C MS Al–Si alloys.

#### Confirmation of abnormal decrease of Si particle size

**Figure 3:**
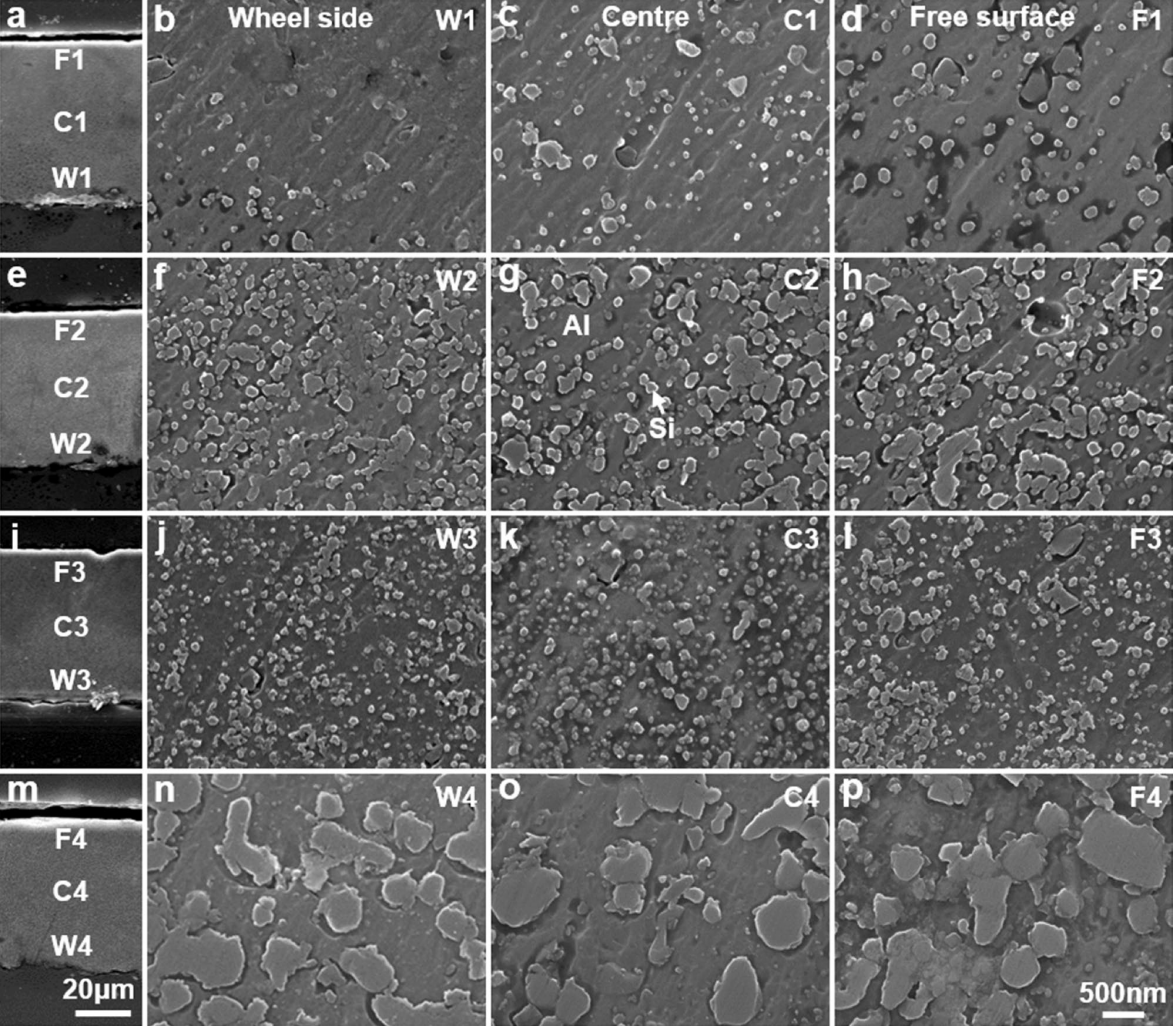
Cross-section SEM morphology of (**a**–**d**) Al–3at.%Si, (**e**–**h**) Al–7at.%Si, (**i**–**l**) Al–12.2at.%Si and (**m**–**p**) Al–20at.%Si melt-spinning alloy ribbons rapid solidified from melts at 1100 °C: (**a**, **e**, **i**, **m**) low magnification (×1 k) cross-section morphology, and high magnification (×30 k) morphology of Si particles (**b**, **f**, **j**, **n**) near the wheel side, (**c**, **g**, **k**, **o**) in the center, and (**d**, **h**, **l**, **p**) near the free surface of the melt-spinning alloy ribbons.

During the MS process, the cooling rate decreases gradually from the wheel side to the free surface of the MS alloy ribbons, which results in the gradual increase of the size of phases across the cross-section of the MS alloy ribbons from the while side to the free surface^27,28^. Therefore, the size of Si particles across the whole cross-section (near the wheel side, in the center and near the free surface) of the 1100 °C MS Al–3at.%Si, Al–7at.%Si, Al–12.2at.%Si and Al–20at.%Si alloy ribbons was studied, to confirm the abnormal decrease of Si particle size in the 1100 °C MS Al–12.2at.%Si alloy. Figure 3a–d,e–h,i–l,m–p show the detail cross-section SEM morphology of the 1100 °C MS Al–3at.%Si, Al–7at.%Si, Al–12.2at.%Si and Al–20at.%Si alloy ribbons, respectively. From the low magnification (×1 k) cross-section morphology shown in Fig. 3a,e,i,m, the thickness of the 1100 °C MS Al–3at.%Si, Al–7at.%Si, Al–12.2at.%Si and Al–20at.%Si alloy ribbons is ~ 55 μm. Figure 3b,f,j,n, Fig. 3c,g,k,o and Fig. 3d,h,l,p show the high magnification (×30 k) morphology of Si particles near the wheel side, in the center and near the free surface of the MS alloy ribbons, respectively. The size of Si particles in the 1100 °C MS Al–Si alloys increases gradually across the cross-section from the wheel side to the free surface due to the decreasing cooling rate. With the increase of Si content, the size of Si particles across the whole cross-section of the 1100 °C MS Al–3at.%Si, Al–7at.%Si, Al–12.2at.%Si and Al–20at.%Si alloy ribbons first increases, then decreases abnormally at Al–12.2at.%Si, after increases again. Thus the abnormal decrease of Si particle size at the 1100 °C MS Al–12.2at.%Si alloy is the fact across the whole cross-section of the 1100 °C MS Al–12.2at.%Si alloy, and it is not a fluke by loosely taking the size of finer Si particles near the wheel side of the 1100 °C MS Al–12.2at.%Si alloy while taking the size of larger Si particles far from the wheel side of the other 1100 °C MS alloys.

**Figure 4:**
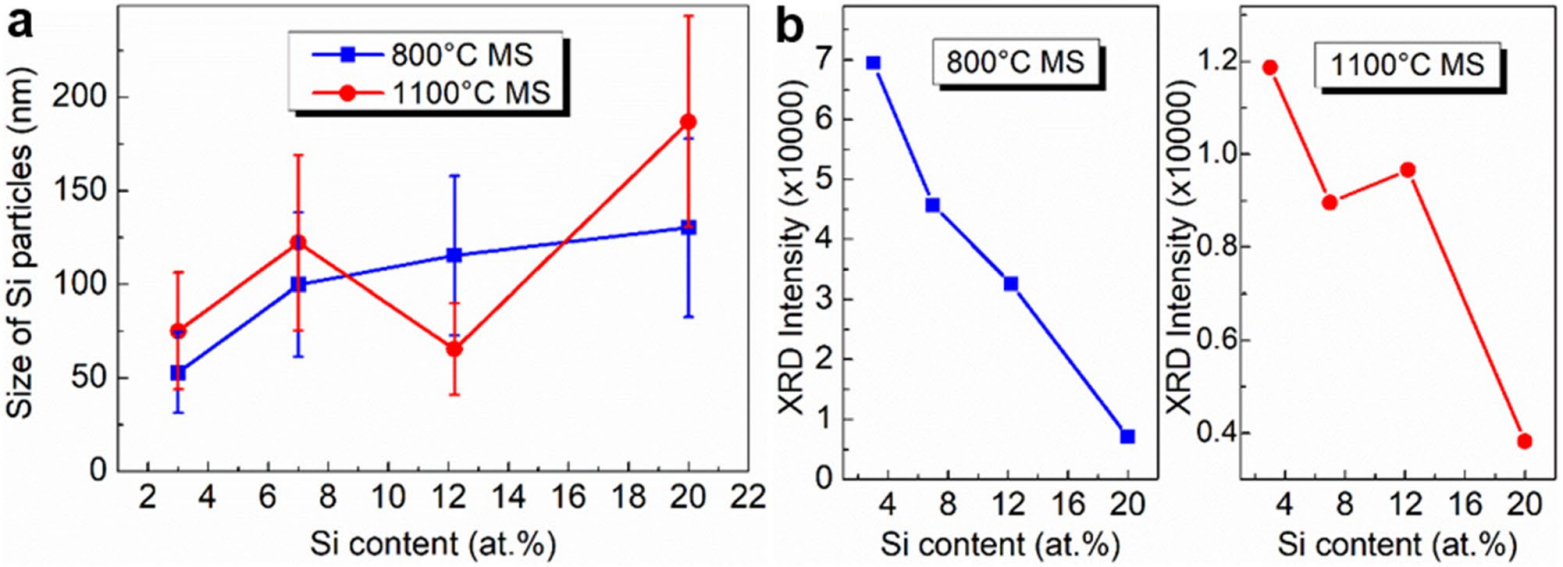
Abnormal decrease of Si particle size in 1100 °C melt-spinning Al–12.2at.%Si alloy. (**a**) Evolution of statistical mean size of Si particles in the investigated 800 °C and 1100 °C melt-spinning Al–Si alloys versus Si content; (**b**) Evolution of the XRD intensity of the Al(111) main peak of the investigated 800 °C and 1100 °C melt-spinning Al–Si alloys versus Si content.

For consistency, the medium magnification (×15 k) SEM micrographs taken from the center of the cross-section were used for the statistics of the size of Si particles in the investigated MS Al–Si alloy ribbons. Figure 4a presents the evolution of the statistical mean size of Si particles in the 800 °C and 1100 °C MS Al–Si alloys versus Si content. All of the investigated MS Al–Si alloys show the increase in Si particle size with the increase of both Si content and initial MS melt temperature, except for the 1100 °C MS Al–12.2at.%Si alloy that shows an abnormal decrease in Si particle size. Figure 4b shows the XRD intensity of the Al(111) main peak of all the investigated MS Al–Si alloys, basing on the XRD patterns shown in Fig. 1. For 800 °C MS Al–Si alloys, with increasing Si content, the Al(111) XRD intensity decreases monotonically, which is consistent with the monotonic increase of Si phase in the alloys observing by SEM. For 1100 °C MS Al–Si alloys, with increasing Si content, the Al(111) XRD intensity decreases in a non-monotonic way, and the abnormal increase of the Al(111) XRD intensity at Al–12.2at.%Si is self-consistent with the abnormal decrease of Si particle size under this condition observing by SEM. The self-consistent SEM and XRD analysis of the 800 °C and 1100 °C MS Al–3at.%Si, Al–7at.%Si, Al–12.2at.%Si and Al–20at.%Si alloys confirms the abnormal decrease of Si particle size and the abnormal increase of Si particle number in the 1100 °C MS Al–12.2at.%Si alloy, which demonstrates the disruption of Si-rich microstructure in the Al–12.2at.%Si alloy melt with the increase of melt temperature from 800 to 1100 °C.

### Small angle neutron scattering of Al–12.2at.%Si alloy melt

Small angle X-ray scattering (SAXS)^29,30^ and small angle neutron scattering (SANS)^5,31,32^ have been applied to study the micro-heterogeneous structure in solid materials and solutions especially the colloidal suspension system, as the measured intensity of small angle scattering (SAS) is proportional to the contrast that is given by the difference in the scattering length density between the micro-heterogeneous structure and the matrix, and there is SAS signal for the micro-heterogeneous system, while there is no SAS signal for the homogeneous system without micro-heterogeneous structure embedded in the matrix^33,34^. Considering the abnormal decrease of Si particle size in the 1100 °C MS Al–12.2at.%Si alloy, SANS was applied to further explore the microstructure evolution in the Al–12.2at.%Si alloy melt.

**Figure 5:**
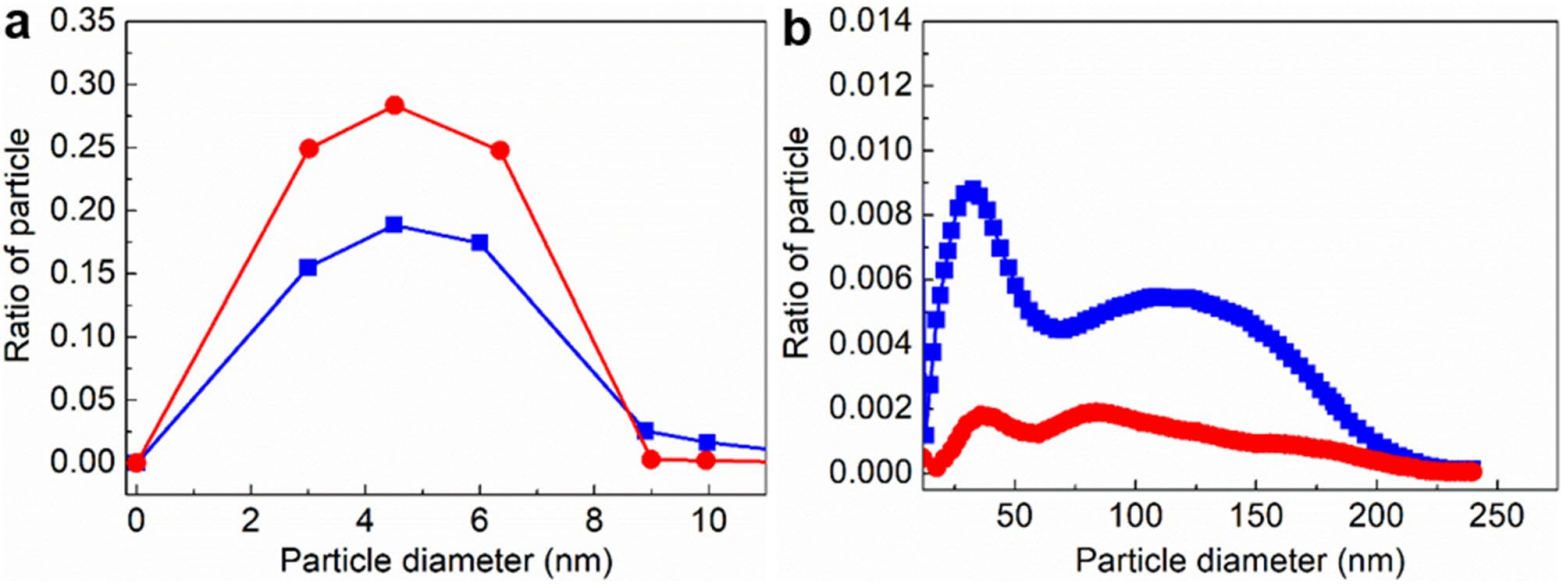
Small angle neutron scattering results showing the distribution of the Si-rich microstructure in the Al–12.2at.%Si alloy melt at 800 °C (blue curve) and 1100 °C (red curve). (**a**) Distribution of small Si-rich microstructure bellow 10 nm, (**b**) Distribution of large Si-rich microstructure above 10 nm.

Figure 5 shows the size distribution of the micro-heterogeneous structure in the Al–12.2at.%Si alloy melt at 800 °C and 1100 °C measuring by SANS, and the nanoscale micro-heterogeneous structure in the Al–12.2at.%Si alloy melt comes from the aggregation of Si atoms, as it has the contrast difference with the Al-rich melt matrix under SANS. The Si-rich micro-heterogeneous structure in the Al–12.2at.%Si alloy melt exists in two size families, i.e., large quantities of small Si-rich micro-heterogeneous structure ranging between 0 and 10 nm and small quantities of large Si-rich micro-heterogeneous structure ranging between 10 and 240 nm. Large quantities of small (0–6 nm) micro-heterogeneous structure was measured in the Sn–26.1at.%Pb alloy melt by SANS^5^, therefore the size of the micro-heterogeneous structure in alloy melts depends on the alloy system. With the increase of the Al–12.2at.%Si melt temperature from 800 to 1100 °C, the proportion of the small (0–10 nm) Si-rich micro-heterogeneous structure in the alloy melt increases, while the proportion of the large (10–240 nm) Si-rich micro-heterogeneous structure in the alloy melt decreases significantly, which further confirms the disruption of large Si-rich micro-heterogeneous structure in the Al–12.2at.%Si alloy melt into small Si-rich micro-heterogeneous structure under the high temperature of 1100 °C.

### TEM analysis of MS Al–Si alloys

**Figure 6:**
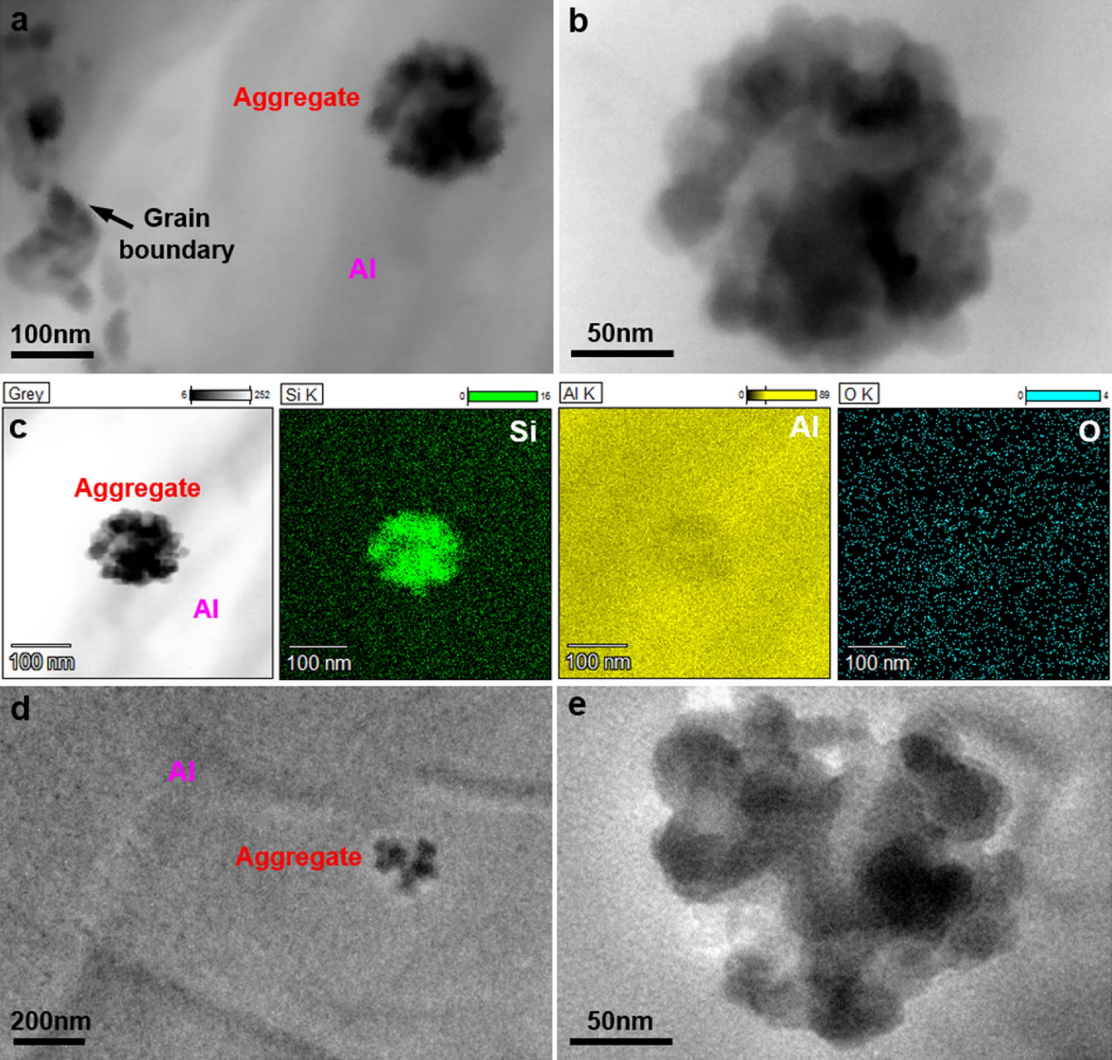
TEM images showing the Si-rich particle aggregates in the Al grains of the melt-spinning Al–Si alloys. (**a**) Scanning TEM image of the Si-rich particle aggregate and (**b**) its magnification in the 800 °C melt-spinning Al–3at.%Si alloy; (**c**) Scanning TEM composition mapping of the Si-rich particle aggregate in (**a**) and (**b**); (**d**) Bright-field TEM image of the Si-rich particle aggregate and (**e**) its magnification in the 800 °C melt-spinning Al–12.2at.%Si alloy.

The above mentioned MS and SANS study demonstrates the Si-rich micro-heterogeneous structure in Al–12.2at.%Si alloy melt at 800 °C, so the 800 °C MS Al–12.2at.%Si alloy was further studied by TEM. Among the eight MS conditions, Si particles in the 800 °C MS Al–3at.%Si alloy are the smallest and closest to the Si-rich micro-heterogeneous structure in the molten state, therefore the 800 °C MS Al–3at.%Si alloy was also analyzed by TEM. Si-rich particle aggregates were observed in the Al grains of the 800 °C MS Al–3at.%Si and Al–12.2at.%Si alloy under TEM, as shown in Fig. 6. Figure 6a presents the scanning TEM (STEM) image of a particle aggregate (160 nm) in the Al grain of the 800 °C MS Al–3at.%Si alloy, and the magnification (Fig. 6b) shows that the particle aggregate in Fig. 6a comprises multiple small particles with the sizes smaller than 30 nm. Figure 6c presents the STEM composition mapping of the particle aggregate shown in Fig. 6a,b and the Al matrix surrounding the particle aggregate, the enrichment of Si and the infertility of Al can be found in the area of the particle aggregate when compared to the surrounding Al matrix, while the concentration of oxygen (O) in the particle aggregate is uniformly the same as that in the surrounding Al matrix, and the concentration of O is much lower than that of Al and Si, which demonstrates that the particle aggregate is not the Al-based or Si-based oxide and the particle aggregate is rich in Si. The uniform appearance of trace O in the STEM mapping was due to the inevitable oxidation of the surface of the TEM sample. Figure 6d shows the bright-field TEM image of a Si-rich particle aggregate in the Al grain of the 800 °C MS Al–12.2at.%Si alloy, and the particle aggregate also comprises multiple small nanoscale Si-rich particles below 50 nm, while the size of small Si-rich particles in the particle aggregate of the 800 °C MS Al–12.2at.%Si alloy is slightly larger than that of the 800 °C MS Al–3at.%Si alloy.

## Discussion

It is understandable that the size of Si particles in the 800 °C and 1100 °C MS Al–3at.%Si, Al–7at.%Si, Al–12.2at.%Si and Al–20at.%Si alloys increases with the increase of both Si content and initial MS melt temperature, due to the increasing growth tendency of Si phase under higher Si content and the decreasing cooling rate under higher initial MS melt temperature. However, Si particles in the 1100 °C MS Al–12.2at.%Si alloy show an abnormal decrease in Si particle size together with an abnormal increase of Si particle number, and this clearly demonstrates the disruption of Si-rich microstructure in the Al–12.2at.%Si alloy melt under the high temperature of 1100 °C. The decrease of the ratio of large Si-rich micro-heterogeneous structure and the increase of the ratio of small Si-rich micro-heterogeneous structure under SANS measurement further confirm that the large (10–240 nm) Si-rich micro-heterogeneous structures in the Al–12.2at.%Si alloy melt disrupt into the small (0–10 nm) Si-rich micro-heterogeneous structures with the increase of melt temperature from 800 to 1100 °C. The microstructure evolution with temperature was sporadically evidenced in other systems such as the In-Sn80 and the La_50_Al_35_Ni_15_ alloy melts^4,7^.

The TEM analysis in Fig. 6 shows the entrapment of Si-rich particle aggregates in the Al grains of the 800 °C MS Al–12.2at.%Si alloy. According to the thermal conduction model^35^ of the MS process, the mean cooling rate of the 800 °C MS here was calculated as ~ 2.79 × 10^6^ °C/s, and the Si particle aggregates entrapped in the Al grains had limited time to grow during MS. In addition, the size of the Si-rich particle aggregates entrapped in the Al grains of the 800 °C MS Al–12.2at.%Si alloy agrees with the large (10–240 nm) Si-rich micro-heterogeneous structure in the Al–12.2at.%Si alloy melt measured by SANS. From Fig. 6, the size of small Si-rich particles in the particle aggregate of the 800 °C MS Al–12.2at.%Si alloy is slightly larger than that of the 800 °C MS Al–3at.%Si alloy, which indicates the inevitable growth of the small Si-rich particles in the particle aggregate under the super high cooling rate of the MS process, and the size of the small Si-rich particles in the particle aggregate of the MS alloy fits with the small (0–10 nm) Si-rich micro-heterogeneous structure in the Al–12.2at.%Si alloy melt measured by SANS, when considering the growth effect during MS. Furthermore, the Si-rich particle aggregate can be determined not precipitating out during the MS process, as the precipitation phase is normally precipitated out in the form of single particle rather than aggregate, and the Si-rich particle aggregate entrapped in the Al grain of the 800 °C MS Al–12.2at.%Si alloy is close to its original state in the alloy melt. Moreover, hardly were Si-rich particle aggregates observed in the Al grains of the 1100 °C MS Al–12.2at.%Si alloy, which supports the disruption of large Si-rich aggregates in the Al–12.2at.%Si alloy melt at 1100 °C. Therefore, the large (10–240 nm) Si-rich micro-heterogeneous structures in the Al–12.2at.%Si alloy melt are probably Si-rich aggregates comprising multiple small (0–10 nm) Si-rich micro-heterogeneous structures, due to the agglomeration effects^36^ of nanostructures, and the large Si-rich aggregates in the Al–12.2at.%Si alloy melt might be significantly disrupted into multiple small Si-rich microstructures at 1100 °C. The degrees of superheat of the Al–3at.%Si, Al–7at.%Si, Al–12.2at.%Si and Al–20at.%Si alloys are 459 °C, 485 °C, 523 °C and 406 °C, respectively, at 1100 °C. The superheat of the eutectic Al–12.2at.%Si alloy is the largest among the four investigated Al–Si alloys at the same MS temperature of 1100 °C, and the superheat of 523 °C is sufficient for the significant disruption of Si-rich microstructure in the Al–12.2at.%Si alloy melt, which results in the abnormal decrease of Si particle size in the 1100 °C MS Al–12.2at.%Si alloy. It can be estimated that the Si-rich microstructure in the other three Al–Si alloys can also be significantly disrupted if the superheat is sufficient, and the sufficient superheat for the significant disruption of Si-rich microstructure in the other three Al–Si alloys might be 523 °C or even higher, due to the higher liquidus temperatures and atomic binding in the other three Al–Si alloys than that of the eutectic Al–12.2at.%Si alloy. Nanoscale Si particles were observed absorbing on the surface of the AlB_2_ nucleation particles for the grain refinement of Al–Si alloys^37^, and these nanoscale Si particles might be originated from the small (0–10 nm) Si-rich micro-heterogeneous structures in the Al–Si alloy melts.

**Figure 7:**
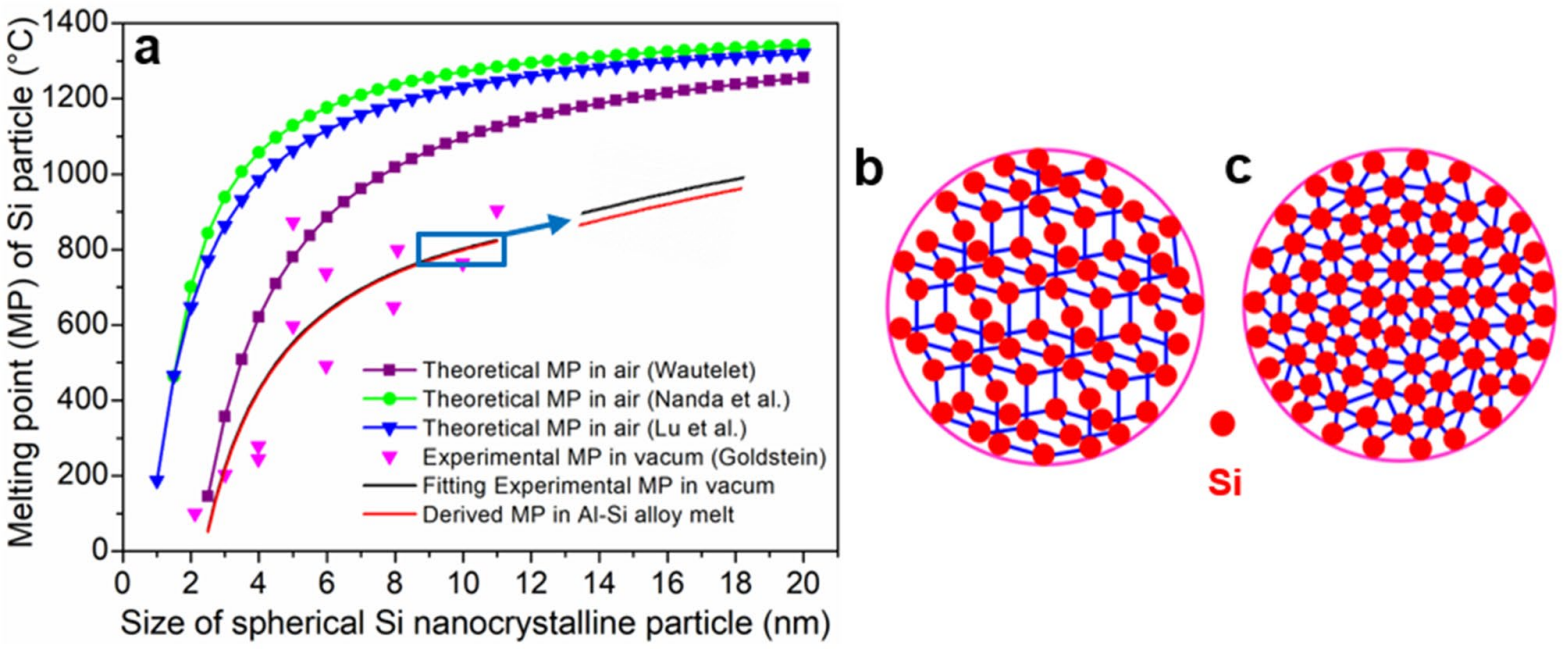
Existing state of the Si-rich microstructure in the Al–12.2at.%Si alloy melt. (**a**) Melting points of solid state spherical Si nanocrystalline particle versus size under various atmospheres; (**b**) Schematic diagram showing the solid state spherical Si nanocrystalline particle with crystalline structure; (**c**) Schematic diagram showing the droplet state Si-rich microstructure in Al–12.2at.%Si alloy melt with non-crystalline structure.

Considering that the melting points of nanoparticles have size effects^38,39^, the melting points of spherical pure Si nanocrystals in the atmosphere of Al–12.2at.%Si alloy melt were determined, to reveal the existing state of the Si-rich micro-heterogeneous structures in the Al–12.2at.%Si alloy melt. Wautelet^40^^,^ Nanda et al.^41^ and Lu et al.^42^ proposed different theoretical models estimating the melting point dependence of spherical nanocrystals versus size. For spherical pure Si nanocrystals, the theoretical melting points given by Wautelet’s model were relatively close to experimental values given by Goldstein^43^ (Fig. 7a). However, a correction factor k (0.785) needs to be added into Wautelet’s theoretical model, to fit the experimental melting points of pure Si nanocrystals in the atmosphere of vacuum:

1 $$ {\text{T}}_{{{\text{mv}}}} = {\text{kT}}_{0} \left( {1 - \frac{\upbeta }{d}} \right)\quad (d > 10{\text{d}}_{0} ) $$

where T_0_ (1687 K) is the melting point of bulk Si, β (1.88 nm) is a constant, *d* is the diameter of the Si nanocrystal, and d_0_ (0.235 nm) is the interatomic distance in crystalline Si.

According to the Clapeyron equation, melting point changes with pressure. Based on Eq. (1), the real melting point of spherical pure Si nanocrystals in the atmosphere of air is:

2 $$ {\text{T}}_{{{\text{ma}}}} = {\text{kT}}_{0} \left( {1 - \frac{\upbeta }{d}} \right)\left( {1 + \frac{{{\text{P}}_{0} \Delta_{\upalpha }^{\upbeta } V_{{\text{m}}} }}{{\Delta_{\upalpha }^{\upbeta } {\text{H}}_{{\text{m}}} }}} \right)\quad (d > 10{\text{d}}_{0} ) $$

where P_0_ is the standard atmospheric pressure (1.01325 × 10^5^ Pa), $\Delta_{{\upalpha }}^{{\upbeta }} H_{{\text{m}}}$ (50.21 kJ**·**mol^−1^) is the molar melting heat of Si, and $\Delta_{{\upalpha }}^{{\upbeta }} V_{{\text{m}}}$(− 1.131 cm^3^ mol^−1^) is the change of molar volume before and after the melting of Si.

In Al–12.2at.%Si alloy melt, the spherical pure Si nanocrystals will suffer from an extra surface tension. Based on the Clapeyron equation and Eq. (2), the real melting points of spherical pure Si nanocrystals in the atmosphere of Al–12.2at.%Si alloy melt are then:

3 $$ T_{{{\text{mm}}}} = {\text{kT}}_{0} \left( {1 - \frac{{\upbeta }}{d}} \right)\left( {1 + \frac{{{\text{P}}_{0} {\Delta }_{{\upalpha }}^{{\upbeta }} V_{{\text{m}}} }}{{{\Delta }_{{\upalpha }}^{{\upbeta }} H_{{\text{m}}} }}} \right)\left( {1 + \frac{{4\sigma {\Delta }_{{\upalpha }}^{{\upbeta }} V_{{\text{m}}} }}{{d{\Delta }_{{\upalpha }}^{{\upbeta }} H_{{\text{m}}} }}} \right)\;\;(d > 10{\text{d}}_{0} ) $$

where *σ* (0.35241 J m^-2^)^44^ is the surface tension.

According to Eq. (3), the melting points of spherical pure Si nanocrystalline particles in the atmosphere of Al–12.2at.%Si alloy melt were calculated, as shown by the red curve in Fig. 7a, which are ~ 4–6 °C lower than that in the atmosphere of air. The melting points of the pure Si nanocrystalline particles with diameters of 3, 4, 5, 6, 7, 8, 9 and 10 nm were derived as 216, 423, 548, 631, 691, 736, 771 and 799 °C, respectively, in the atmosphere of Al–12.2at.%Si alloy melt. The melting point of Si-rich micro-heterogeneous structure is lower than that of the pure Si nanocrystalline particle with the same size, in the atmosphere of Al–12.2at.%Si alloy melt. Therefore, the small (0–10 nm) Si-rich micro-heterogeneous structures in the 800 °C Al–12.2at.%Si alloy melt exist in the state of droplet with non-crystalline structure (Fig. 7c), rather than the state of solid crystals with crystalline structure (Fig. 7b), as their melting points are lower than the melt temperature. Correspondingly, the large (10–240 nm) Si-rich aggregates comprising multiple small (0–10 nm) Si-rich micro-heterogeneous structures in the Al–12.2at.%Si alloy melt exist in the state of droplet aggregates. The state of droplet in turn supports the spherical shape of the Si-rich micro-heterogeneous structures in Al–12.2at.%Si alloy melt, due to the surface tension.

Earlier a thermodynamic metastable colloidal model^45^ of liquid metallic solutions was proposed to explain the coexistence of micro-heterogeneous droplets in alloy melts, and the nanoscale micro-heterogeneous droplets in Sn–Pb alloy melt^5^ could be thermodynamically metastable by a thin transition layer with a thickness of ~ 0.3 nm^45^, to reach the local minimum of the free energy of the system. Here the droplet state Si-rich micro-heterogeneous structure is possibly metastable in the Al–12.2at.%Si alloy melt via a thin transition layer, which provides the thermodynamic base for the disruption of the Si-rich micro-heterogeneous structure in the Al–12.2at.%Si alloy melt with the increase of melt temperature from 800 to 1100 °C.

## Conclusion

In summary, we provide experimental evidence for the disruption of Si-rich micro-heterogeneous structure in the engineering-lightweight Al–12.2at.%Si alloy melt above liquidus temperature. The size of Si particles in 800 °C and 1100 °C MS Al_1−x_Si_x_ (x = 0.03, 0.07, 0.122, 0.2) alloys increases with the increase of both Si content and initial MS melt temperature, except for the 1100 °C MS Al–12.2at.%Si alloy that shows an abnormal decrease in Si particle size together with an abnormal increase in Si particle number, which demonstrates the disruption of Si-rich microstructure in Al–12.2at.%Si alloy melt at 1100 °C. Small angle neutron scattering experiment further verifies that large quantities of small (0–10 nm) Si-rich micro-heterogeneous structures and small quantities of large (10–240 nm) Si-rich micro-heterogeneous structures exist in Al–12.2at.%Si alloy melt, and the large Si-rich micro-heterogeneous structures in the Al–12.2at.%Si alloy melt disrupt into small Si-rich micro-heterogeneous structures with increasing of melt temperature from 800 °C to 1100 °C. Si-rich particle aggregates comprise multiple small Si-rich particles were observed entrapping in the Al grains of the 800 °C MS Al–12.2at.%Si alloy, which agree in size with the large Si-rich micro-heterogeneous structures in the Al–12.2at.%Si alloy melt, and this indicates that the large (10–240 nm) Si-rich micro-heterogeneous structures in the Al–12.2at.%Si alloy melt are probably Si-rich aggregates comprising multiple small (0–10 nm) Si-rich micro-heterogeneous structures. The method of MS from different initial temperatures and compositions in cooperation with the small angle neutron scattering measurement presented in this work also provides a pathway for the exploration of microstructures in other high temperature alloy melts.

## Materials and methods

A series of high purity hypoeutectic Al–3at.%Si and Al–7at.%Si, eutectic Al–12.2at.%Si and hypereutectic Al–20at.%Si binary Al–Si alloys were prepared by the arc-melting of the high purity Al (5N) and Si (5N). The alloys were remelted three times under the inert Ar atmosphere, to ensure the composition homogenization of the prepared high purity Al–Si alloys. The prepared high-purity Al–3at.%Si, Al–7at.%Si, Al–12.2at.%Si and Al–20at.%Si alloy melts were rapidly solidified with super-high cooling rate (~ 10^6^ °C/s) into thin ribbons by the MS^27,46,47^ method under high-vacuum (3.3E-5 Pa) condition. In the melt-spinner, alloy charges of ~ 3 g were melted in a Ø 12 mm crucible with 5 mm × 0.5 mm orifice and ejected by argon onto a rotating copper wheel with the linear velocity of 32.8 m·s^−1^. All alloy melts were MS from two different initial melt temperatures, 800 °C and 1100 °C. The melt temperatures during MS were measured and controlled with an accuracy of ±6 °C. The resultant MS Al–Si alloy ribbons were ~ 5 mm wide and ~ 55 μm thick.

The prepared high purity Al–12.2at.%Si alloy melt was measured by SANS at 800 °C and 1100 °C. The sample for SANS was contained in a single crystalline sapphire container with a diameter of 10 mm and a wall thickness of 50 μm. The incident neutron wavelength was chosen as 8 Å, to avoid the multiple scattering in the container walls. After rising to the melt temperatures of 800 °C and 1100 °C, the alloy melt was held for 1 h before SANS measurement, and the SANS measurement time was 1 h afterwards.

SEM, XRD and TEM were used to characterize the microstructure of the prepared MS Al–Si alloy ribbons. The MS alloy ribbons for SEM investigation were polished and etched by 0.5 vol.% HF. SEM observation was performed on JSM-7001F. The XRD measurements were carried out in a Rigaku H2500 diffractometer using Cu Ka1 radiation at 40 kV and 20 mA in the 2*θ* range from 25° to 85°. The MS alloy ribbons for TEM investigation were ion-beam milled under a constant preparation temperature of ~ − 10 °C. The Tecnai G^2^ F20 and JEOL-2100 TEM were used at 200 kV for bright-field imaging, scanning TEM imaging and composition mapping.

For consistency and accuracy, the medium magnification (×15k) SEM micrographs taken from the center of the cross-section of the MS Al–Si alloy ribbons were used for the statistics of the size and distribution of Si particles in each of the MS alloys. The statistics was conducted in the software Nano Measurer 1.2.5 by measuring all the sizes of Si particles in the micrographs. The particle size distribution under SANS measurement was determined using the Generalized Indirect Fourier Transformation method as developed in the GIFT computer code^48^.

## Data Availability

The data that support the findings of this study are available from the corresponding authors upon reasonable request.
